# A Robust and Energy-Efficient Transport Protocol for Cognitive Radio Sensor Networks

**DOI:** 10.3390/s141019533

**Published:** 2014-10-20

**Authors:** Shelly Salim, Sangman Moh

**Affiliations:** Department of Computer Engineering, Chosun University, 309 Pilmun-daero, Dong-gu, Gwangju 501-759, Korea; E-Mail: shellysalim22@gmail.com

**Keywords:** cognitive radio sensor network, transport protocol, energy efficiency, network lifetime, event-detection delay

## Abstract

A cognitive radio sensor network (CRSN) is a wireless sensor network in which sensor nodes are equipped with cognitive radio. CRSNs benefit from cognitive radio capabilities such as dynamic spectrum access and transmission parameters reconfigurability; but cognitive radio also brings additional challenges and leads to higher energy consumption. Motivated to improve the energy efficiency in CRSNs, we propose a robust and energy-efficient transport protocol (RETP). The novelties of RETP are two-fold: (I) it combines distributed channel sensing and channel decision with centralized schedule-based data transmission; and (II) it differentiates the types of data transmission on the basis of data content and adopts different acknowledgment methods for different transmission types. To the best of our knowledge, no transport layer protocols have yet been designed for CRSNs. Simulation results show that the proposed protocol achieves remarkably longer network lifetime and shorter event-detection delay compared to those achieved with a conventional transport protocol, while simultaneously preserving event-detection reliability.

## 1. Introduction

A wireless sensor network (WSN) is a wireless network that consists of a large number of sensor nodes that can be applied for environment monitoring, invasion detection, disaster aid, and various other fields [1]. The lifetime of a WSN depends mostly on sensor node batteries. Sensor nodes can preserve their energy in order to extend the network lifetime by adopting energy-efficient protocols. The sensed data are transmitted to a sink node through wireless links, making the data vulnerable to error. In order to transmit the data in a reliable manner, the use of an efficient transport protocol is essential. Especially, in WSNs, it is necessary to design a transport protocol that ensures energy efficiency while preserving reliability.

All types of the wireless technologies use a portion of radio frequency spectrum to perform data transmission. The radio frequency spectrum is a limited resource and it is allocated to several licensed parties. The unlicensed radio spectrum is usually congested, whereas the licensed radio spectrum is often underutilized. Therefore, the licensed radio spectrum can be better utilized by allowing non-licensed users to transmit as long as they do not disturb the data transmission of licensed users. To enable non-licensed or secondary users (SUs) to switch and transmit their data in the licensed spectrum, a special kind of transmission radio called cognitive radio has been proposed [2]. The fundamental capabilities of cognitive radio are spectrum sensing, spectrum mobility, and reconfiguration. Because the cognitive radio has different characteristics and ways of operations compared with the existing wireless radio, the networking layers (physical layer, medium access layer, network layer, transport layer, and application layer) need modifications to suit cognitive radio network environments [3]. The most important consideration of SUs is to avoid interference with licensed or primary users (PUs) on the licensed spectrum. The WSN in which the sensor nodes are equipped with cognitive radio is called a cognitive radio sensor network (CRSN) [4].

Although cognitive radio technology has received a lot of attention recently, studies on designing transport protocols to suit cognitive radio networks are relatively few in number. A transport protocol provides congestion control and reliability [5]. Congestion control is necessary to avoid network congestion by adjusting the transmission rate or by adopting other strategies. Reliability is concerned with the provision of stable and error-free data transmission. When a data transmission event occurs, the data sender should be able to confirm that the receiver has received the data correctly. The receiver should be able to notify the data sender if it does not receive the transmitted data or if the received data are erroneous. A common method to ensure reliability is by requiring the receiver to send an acknowledgment (ACK) packet to the sender on correct reception of data.

Generally, transport protocols used in WSNs can be categorized depending on whether they focus on reliable transmission or on congestion control. Event-to-sink reliable transport (ESRT) [6], reliable multi-segment transport (RMST) [7], and “pump-slowly, fetch-quickly” (PSFQ) transport [8] are some protocols that have been proposed to achieve reliable transmission. The ESRT protocol reduces energy consumption by its low complexity but its transmission speed depends on the environment. The RMST protocol has a drawback of decreased energy efficiency because of its high complexity, but it has an advantage of highly efficient memory management. The PSFQ protocol reduces the transmission speed considerably, but it quickly restores reliability. The representative protocols for congestion control are the congestion detection and avoidance (CODA) protocol [9] and the sensor transmission control protocol (STCP) [10]. The CODA protocol controls network congestion by allowing the nodes to control the transmission rate after congestion is detected. The drawback of this protocol is that the loss of the ACK packet makes the transmission rate, delay, and response time longer because of network congestion.

The existing works on transport layer protocols for WSNs are not suitable for CRSNs mainly because they do not consider the aspect of dynamic spectrum access [11,12]. Several transport protocols have been proposed for general cognitive radio wireless *networks* and cognitive radio *ad hoc* networks. These transport protocols do not consider the resource limitations of the sensor nodes, especially the energy constraint. One of the frequently-cited transport protocols is the transport protocol for cognitive radio *ad hoc* networks (TP-CRAHN) [13]. TP-CRAHN adapts TCP to suit the cognitive radio environment by creating six states, including spectrum sensing and spectrum change. A continuation work of TP-CRAHN can be found in [14]. To the best of our knowledge, no transport layer protocols have yet been designed for CRSNs.

In this paper, we propose a transport layer protocol called the robust and energy-efficient transport protocol (RETP) for CRSNs. The motivation for developing RETP is to improve the network lifetime of CRSNs while achieving low event-detection delay without any degradation of reliability level. The main features of RETP are (I) combination of distributed channel sensing and channel decision with centralized schedule-based data transmission; and (II) differentiation of data transmission types and adoption of acknowledgment methods based on data content. The main contribution of this paper is to provide a transport protocol with high energy-efficiency that leads to prolonged network lifetime and, at the same time, preserve event-detection reliability in CRSNs. Our performance study shows that the proposed RETP not only prolongs network lifetime significantly but also decreases event-detection delay while preserving event-detection reliability compared with the conventional protocol.

The rest of the paper is organized as follows: In Section 2, the features and the algorithms of the proposed RETP are presented in detail. In Section 3, the performance of the proposed RETP is evaluated and compared with that of a conventional transport protocol using computer simulation and the simulation results are discussed. Conclusions drawn are presented in Section 4.

### 2. Robust and Energy-Efficient Transport Protocol (RETP)

In this paper, we propose a robust and energy-efficient transport protocol (RETP) for CRSNs. The underlying CRSN is modeled as follows:

- Each sensor node is equipped with a cognitive radio transmitter.
- Sensor nodes are installed manually following a predetermined topology (not random) and the sink is aware of the location of each sensor node. Fundamentally, RETP can be applied in any topology, including random and clustered topology [15].
- Each sensor node can reach the sink in one hop (by reconfiguring its transmission parameters).
- There is one dedicated common control channel (CCC).
- The CRSN is deployed in an urban environment where PUs are present.
- The CRSN applications being considered are related to the realization of the smart city concept, particularly in terms of disaster avoidance mechanisms such as structural health monitoring, air pollution control, and earthquake/landslide/flood warnings.
- The CRSN network adopts time-division multiplexing in which time is divided into frames and each frame is divided into timeslots. The multiple access method used is code-division multiple access coordinated by the sink.

The features of RETP are:

- There are two operation modes: management mode and data collection mode. In the management mode, sensor nodes perform spectrum management in addition to data collection whereas in data collection mode, sensor nodes perform only data collection. Spectrum management activities include spectrum sensing and spectrum decision, which are enabled by cognitive radio. We did not specify a spectrum sensing method; however, we assume that our proposed work is compatible with general spectrum sensing methods for cognitive radio networks [16,17] or even cooperative spectrum sensing [18].
- Every sensor node determines its operating channel distributively and sends data to the sink according to a specified schedule. This feature provides more accurate spectrum sensing and spectrum decision, as well as saves energy by adopting a coordinated duty cycle.
- The interchange of ACK packets and negative ACK (NACK) packets depends on the sensor data type. This feature ensures the reliability of delay-sensitive data transmission.
- The sensor nodes collect the data and send them to the sink regularly. Thus, the proposed method is suitable for CRSN applications that require regular data collection, but it can also be applied to event-based CRSN.

### 2.1. Operation Modes

In RETP, there are two operation modes: management mode and data collection mode. Initially, the network starts in the management mode, and subsequently the sink manages the next mode based on the condition of the sensor nodes. Each mode is performed during a frame. The management mode includes spectrum management and data transmission activities, whereas the data collection mode consists only of data transmissions. The activities related to data transmission in both the modes are similar.

**Figure 1.**
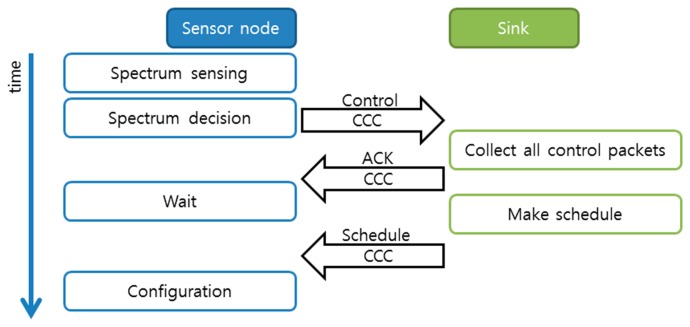
Spectrum management activities (CCC stands for common control channel).

The management mode starts with spectrum management activities. The sensor nodes perform spectrum sensing on the entire spectrum bands assigned. Based on the spectrum sensing result, each sensor node performs spectrum decision to select an operating channel and a back-up channel. The spectrum decision method in our paper is a simple one; that is, the sensor node randomly chooses one of the available spectrums. The current state-of-the-art methods for spectrum decision can be found in [19]. Next, each sensor node reports its preferred operating channel to the sink on the common control channel and waits for further coordination. The sink collects all the control packets containing the operating channels selected by the sensor nodes and constructs a schedule called the sink schedule (S-schedule). The S-schedule contains three elements: time, channel, and reporting nodes. The sink broadcasts the S-schedule on the common control channel and follows this schedule during the data transmission activities. For instance, at time *t_i_*, the sink waits on channel *c_i_* for data transmissions from the sensor nodes that uses *c_i_* as their operating channel. After that, at time *t_i_*_+1_, the sink switches to another channel, *c_j_*, and waits for data transmissions from the sensor nodes. The sink continuously switches the channel and collects the data from the sensor nodes. The sensor nodes receive the S-schedule on the common control channel and create their own schedule. The sensor nodes extract the time information in which the sink is expected to listen on their operating channel. The sensor nodes then go to the sleep state and wake up when they need to perform environment sensing and send the data to the sink according to the S-schedule. The spectrum management activities are illustrated in Figure 1.

**Figure 2.**
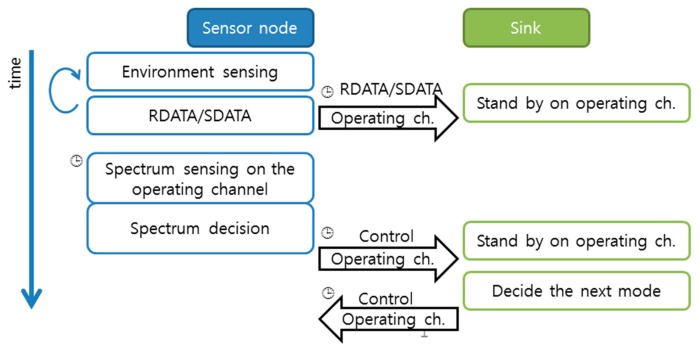
Data transmission activities. The clock image means that the activity is performed by following the S-schedule. (ch. stands for channel.).

After spectrum management activities are completed, the sensor nodes and the sink perform data transmission activities during the remaining time of the management mode (the same frame that starts with spectrum management). The data transmission activities of the sensor nodes and the sink simply follow the S-schedule. The sink switches and listens to different channels and collects the data transmitted by the sensor nodes. The sensor nodes wake up, perform environment sensing (data reading from the environment), and send the data to the sink on their operating channel at predetermined schedules. Once the transmission is completed, the sensor nodes go to the sleep state. The data transmission activities are illustrated in Figure 2.

At the end of data transmission activities, the sensor nodes are required to perform spectrum sensing on their operating channel. Depending on the result of spectrum sensing, each sensor node performs spectrum decision. The possible outcomes of spectrum decision are: (1) the operating channel remains unchanged; (2) owing to the detection of PUs' transmission on the operating channel, the operating channel for the next frame is changed to the back-up channel and the back-up channel set becomes empty; and (3) when the sensor node decides to change its operating channel to the back-up channel but the back-up channel set is empty, the sensor node will request management mode on the next frame to the sink. The outcome of the spectrum decision stage is forwarded to the sink on the common control channel. The sink decides the operation mode for the next frame based on the reported spectrum decision from the sensor nodes. If there is at least one sensor node that requests management mode, then the sink announces the operation mode for the next frame as management mode. Otherwise, the sink creates a new S-schedule, announces that the operation mode for the next frame is data collection mode, and piggybacks the S-schedule with the announcement.

The data collection mode consists of data transmission activities that follow the S-schedule broadcasted by the sink at the end of the previous frame. The S-schedule created by the sink consists of the activities until the end of the current frame that includes spectrum sensing and spectrum decision at the end of the frame.

### 2.2. Interchange of ACK and NACK

There are two types of data: sensitive data (SDATA) and regular data (RDATA). SDATA are the data that reflect critical events, such as the rapid spreading of cracks on a building wall or a bridge, dangerous levels of air pollution/water/temperature, *etc*. Thus, SDATA must be sent to the sink immediately. On the other hand, RDATA are collected periodically for data monitoring. Transmission of RDATA can be postponed if SDATA is present. In the case of SDATA transmission, ACK method is adopted; for RDATA transmission, NACK method is used.

By default, the sink expects regular RDATA transmissions from sensor nodes. The sink anticipates one RDATA transmission during a predetermined period. If the sink receives RDATA successfully, then it stores the data; otherwise, the sink sends a NACK packet to the sensor node whose RDATA is not received and waits for RDATA retransmission on the next scheduled transmission. The sensor node that receives the NACK packet retransmits RDATA on the next transmission schedule (following the S-schedule from the sink). If the sensor node keeps receiving NACK, then it assumes that the current operating channel's quality has been degraded and changes its operating channel or requests management mode on the next frame. As RDATA is not delay-sensitive data, the transmission of RDATA can be postponed.

**Figure 3.**
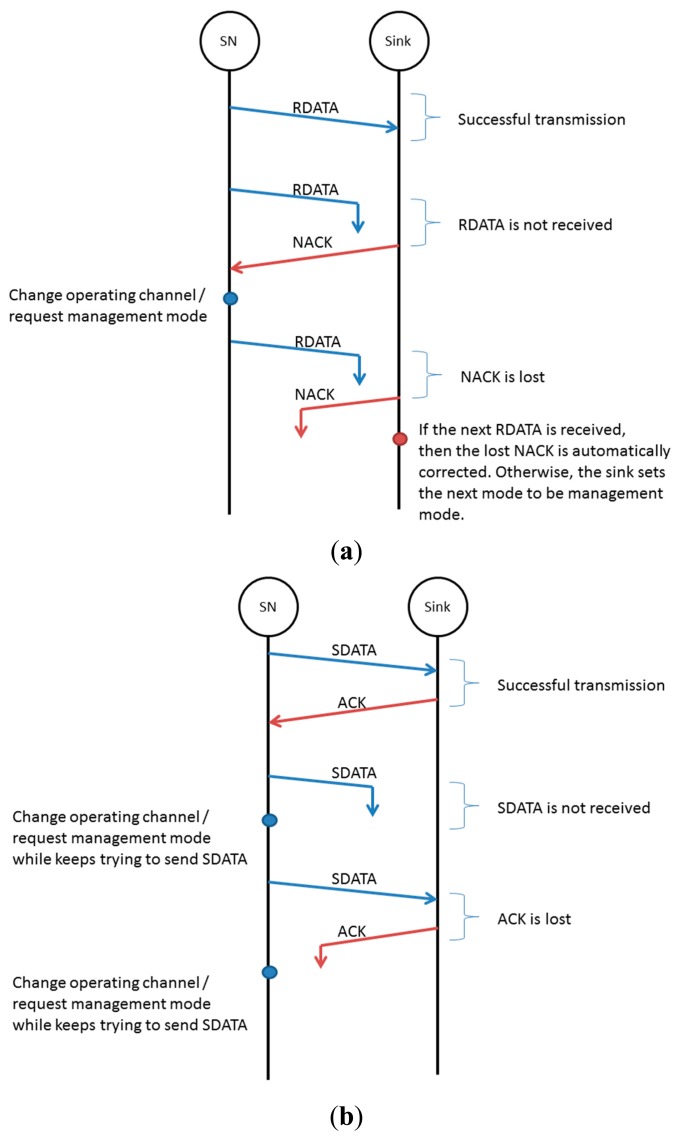
(**a**) NACK method for RDATA and (**b**) ACK method for SDATA.

When the sensor node detects an event in the environment such that its reading exceeds the predetermined threshold, it sends SDATA and waits for an ACK from the sink. If an ACK is not received, the sensor node sends SDATA once more at the next data transmission schedule. If the sensor node still does not receive an ACK, it checks the S-schedule and changes its operating channel to follow the sink's channel until it receives an ACK from the sink. After that, the sensor node changes its operating channel or requests management mode on the next frame. This interchange of ACK and NACK is illustrated in Figure 3.

### 2.3. Analysis of Energy Consumption

In this subsection, we derive the analysis of energy consumption. The energy consumption during data transmission is analyzed first. The data transmission contains the environment sensing data (RDATA or SDATA), which is sent from the sensor nodes to the sink regularly. Then, the energy consumption in the management and data collection modes is comparatively analyzed. Because data collection is the main objective in CRSNs, the number of data collection mode occurrences should be higher than that of management mode occurrences. Furthermore, to preserve energy, the energy consumption in the data collection mode should be lower than that in the management mode. In this analysis, we derive a boundary condition where the energy consumption in the data collection mode is lower than that in the management mode.

#### 2.3.1. Energy Consumption during Data Transmission

In this section, we derive the energy consumption during the transmission of sensed data from the sensor nodes to the sink. As described earlier, there are two types of data: SDATA and RDATA. In each data transmission, either SDATA or RDATA is sent to the sink by following the protocols described in Section 2.2. Hence, the energy consumption during data transmission, *E_TR_*, is derived as follows:

(1) $$E_{TR}=\left(1-q\right)E_{\text{RDATA}}+qE_{\text{SDATA}}$$

where *q* is the probability of SDATA transmission, and *E_RDATA_* and *E_SDATA_* are the energy consumption of RDATA transmission and SDATA transmission, respectively. First, we derive the *E_RDATA_* and *E_SDATA_* and then incorporate them to Equation (1). If *p* is the probability of successful data transmission and *E_TX_* is the energy consumption of transmitting data as well as receiving and decoding data, then by following the protocols of RDATA and SDATA transmission,

(2) $$E_{\text{RDATA}}=\left[p\left(1-p\right)\right]{\left(E_{TX}\right)}^{2}+\left[p^{2}+1-p\right]E_{TX}$$

and

(3) $$E_{\text{SDATA}}=p{\left(E_{TX}\right)}^{2}+(1-p)E_{TX}$$

Hence,

(4) $$E_{TR}=\left[p\left(1-p+pq\right)\right]{\left(E_{TX}\right)}^{2}+\left[\left(1-p\right)-p^{2}\left(1-q\right)\right]E_{TX}$$

If we calculate *E_TR_* using the *E_TX_* value used in the simulation in Section 3, then

(5) $$\text{mean}\left(E_{TR}\right)≅1.25E_{TX}$$

### 2.3.2. Energy Consumption in the Management and Data Collection Modes

To achieve better spectrum selection and lower overhead, the number of management mode occurrences should be lower than that of data collection mode occurrences. This means that there are more data transmissions than management activities. Moreover, to save energy, the energy consumption in the data collection mode (*E_DC_*) should be lower than that in the management mode (*E_M_*). *E_DC_* and *E_M_* can be defined by

(6) $$E_{DC}=(E_{ES}+E_{TR})d_{DC}+E_{LM}$$

and

(7) $$E_{M}=C_{T}E_{SS}+E_{SM}+\left(E_{SS}+E_{TR}\right)d_{M}+E_{LM}$$

respectively, where *E_ES_* is the energy consumption of environment sensing, *E_SS_* is the energy consumption of spectrum sensing, *E_SM_* is the energy consumption of spectrum management in the management mode, *E_LM_* is energy consumption of the last spectrum management performed at the end of a frame, *C_T_* is the total number of channels, *d_DC_* is the number of data collection cycles in the data collection mode, and *d_M_* is the number of data collection cycles in the management mode. Because of the energy consumption values are fixed, both *E_DC_* and *E_M_* greatly depend on *d_DC_* and *d_M_*, respectively. There are three possible conditions between *d_DC_* and *d_M_*:

(1) If *d_DC_* = *d_M_*, then *E_DC_* < *E_M_*.
(2) If *d_DC_* < *d_M_*, then *E_DC_* ≪ *E_M_*.
(3) If *d_DC_* > *d_M_*, then there should be a limitation to make *E_DC_* < *E_M_*.

The duration of a frame is fixed as 100 time slots in our performance evaluation in Section 3. Furthermore, certain activities are occurred determinately and their time consumption is predetermined. Hence,

(8) $$d_{DC}=93/\left(3C_{DC}\right)$$

and

(9) $$d_{M}=\left(87-C_{T}\right)/\left(3C_{M}\right)$$

where *C_T_* is the total number of channels, and *C_DC_* and *C_M_* are the numbers of active channels in the data collection mode and in the management mode, respectively. Furthermore, 1 ≤ *C_DC_* ≤ *C_T_* and 1 ≤ *C_M_* ≤ *C_T_*. Let *E* = *E_ES_* + *E_TR_*, *E′* = *C_T_* *E_SS_* + *E_SM_*, and *E_LM_* be eliminated in both *E_DC_* and *E_M_*, then, to show that *E_DC_* < *E_M_*, ∀ *d_DC_* > *d_M_*, the following equation should be true:

(10) $$\begin{array}{c} E\left(d_{DC}-d_{M}\right)<E\prime ,\;\text{where}\;\left(d_{DC}-d_{M}\right) \end{array}>1.$$

To check the absolute truth of Equation (10), we compare the maximum(*E*(*d_DC_* − *d_M_*)) and minimum(*E′*). We can further analyze this: maximum(*E*(*d_DC_* − *d_M_*)) = *E* × maximum(*d_DC_* − *d_M_*) = *E* × (maximum(*d_DC_*) − minimum(*d_M_*)). The *d_DC_* is maximized when *C_DC_* equals to 1. Hence, *d_DC_* equals to 31 whereas the minimal *d_M_* equals to 1.

Using the same variables used in the simulation in Section 3, *E_ES_* ≒ *E_TX_* and, using the result from Equation (5),

(11) $$E≒2.25E_{TX}$$

Similarly, *E_SS_* ≒ 0.*5E_TX_* and *E_SM_* ≒ 4.67 *E_TX_*. From the setting of *d_M_* = 1 and maximal *C_M_*, we can obtain that *C_T_* equals to 21. Hence,

(12) $$E\prime ≒15.17E_{TX}$$

By substituting Equations (12) and (13) into Equation (10), we found that the statement (10) is not true. This means that ∃ *d_DC_* and *d_M_* such that *E_DC_* < *E_M_*.

Now, we proceed to found the boundary of *d_DC_* and *d_M_*. The boundary of *d_DC_* and *d_M_* can be simplified into the boundary of *C_T_*, *C_DC_* and *C_M_* by referring to Equations (8) and (9). We knew that 1 ≤ *C_DC_* ≤ *C_T_* and 1 ≤ *C_M_* ≤ *C_T_*, ∀ *C_DC_*, *C_M_*, and *C_T_*. First, we evaluate the condition when *C_DC_* = *C_M_*. Using Equations (8) and (9), *d_DC_* > *d_M_*, ∀ *C_DC_*, *C_M_* and *C_T_* > 1. Then, when *C_DC_* ≠ C*C_M_*, by setting minimal *d_DC_* = 2 and maximal *d_M_* happens when *C_M_* = 1, *d_DC_* > *d_M_*, given *d_DC_* ≥ 2 and *C_T_* > 81. However, we only consider the first condition (*C_DC_* = *C_M_*) because, in the simulation in Section 3, we set *C_T_* < 81 and the case of *C_T_* > 81 is rare in the practical situation. Therefore,

(13) $$1\leq C_{T}\leq 80\;\text{and}\;C_{DC}=C_{M}=C,1\leq C\leq C_{T}$$

Thus, we can represent Equations (8) and (9) as

(14) $$d_{DC}=31/C$$

and

(15) $$d_{DC}=\left(87-C_{T}\right)/\left(3C\right)$$

respectively. Also, referring to Equations (11) and (12) and eliminating the term *E_TX_* at both equations, we obtain:

(16) $$E_{DC}=E\times d_{DC}=279/(4C)$$

(17) $$E_{M}=E\times d_{M}=\frac{2.25\left(87-C_{T}\right)}{3C}+0.5C_{T}+4.67$$

Now, by setting *E_DC_* < *E_M_*, we obtain the limit of *C* as

(18) $$C\geq \left\lceil \left(54+9C_{T}\right)/\left(56+6C_{T}\right)\right\rceil$$

which is the boundary condition where the energy consumption in the data collection mode is lower than that in the management mode. When *C_T_* is the maximum of 80, *C* should be ≥ 2. In the simulation, we set *C_T_* to be equal to 30. So, using Equation (18), *C* ≥ 2, which means that the minimum number of active channels in the management and data collection modes is 2. Therefore, if *d_DC_* > *d_M_*, then *E_DC_* < *E_M_* given the boundary of *C* ≥ ⌈(54 + 9*C_T_*)/(56 + 6*C_T_*)⌉, which satisfies the three possible conditions between *d_DC_* and *d_M_*.

## 3. Performance Evaluation

The performance of RETP is evaluated by a computer simulation using MATLAB. The proposed RETP is compared with TP-CRAHN [12]. Originally, TP-CRAHN was developed for cognitive radio *ad hoc* networks. In our performance study, TP-CRAHN is selected as a comparison work because it is one of the earliest and the most cited transport protocol in cognitive radio network environments.

### 3.1. Simulation Environment

**Figure 4.**
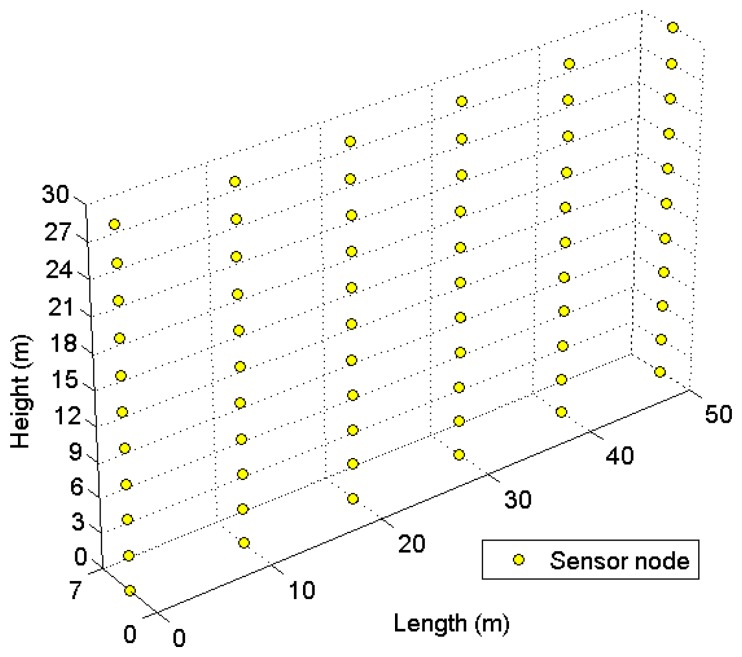
Sensor node deployment.

The sensor nodes are deployed inside a building following a predetermined topology as shown in Figure 4. The application of the CRSN might be structural health monitoring, temperature monitoring, *etc*. The simulation settings are presented in Table 1. The power consumption profiles are adopted from [20,21]. The proposed RETP is compared with TP-CRAHN in terms of the (I) number of alive nodes; (II) delay in event detection; and (III) reliability of event detection. Each simulation is iterated a hundred times.

### 3.2. Number of Alive Nodes

**Figure 5.**
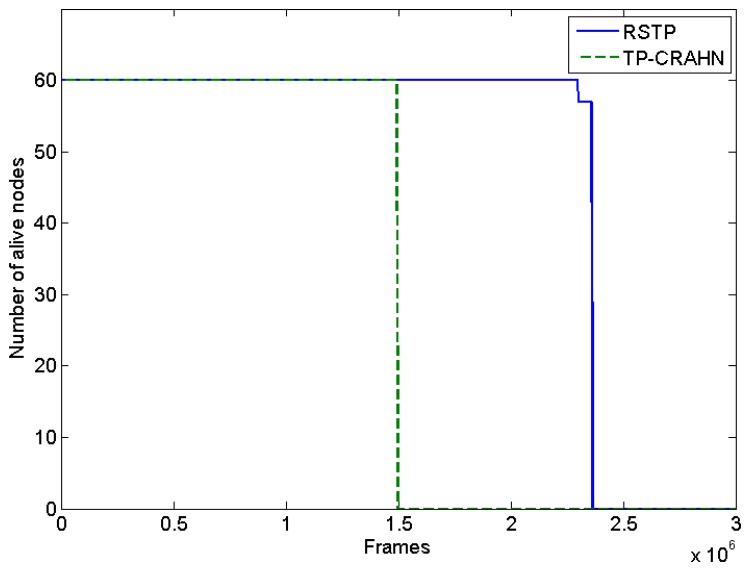
Number of alive nodes.

Figure 5 shows the number of alive nodes per frame during the network active time. As the network topology is predefined and the sensing coverage is not redundant, the exhaustion of even one sensor node means that the CRSN coverage is disrupted. Therefore, we define the network lifetime as the energy depletion of the first sensor node. With this definition, RETP has 53.77% longer lifetime compared with TP-CRAHN. The main reason for low energy consumption in RETP is that it follows the schedule from the sink, according to which a sensor node can go to the sleep state if it has no scheduled activity. In TP-CRAHN, because it was designed for *ad hoc* networks, the sensor nodes are required to perform frequent control channel exchanges and relay data packets. Nevertheless, both protocols perform spectrum decision in a distributive manner, making the spectrum decision results (operating and backup channel selection) more accurate compared to centralized spectrum decision. In this simulation, the frame duration is equal to 2 s; thus, the lifetime of RETP is about 53 days and the lifetime of TP-CRAHN is about 34 days.

### 3.3. Delay in Event Detection

**Figure 6.**
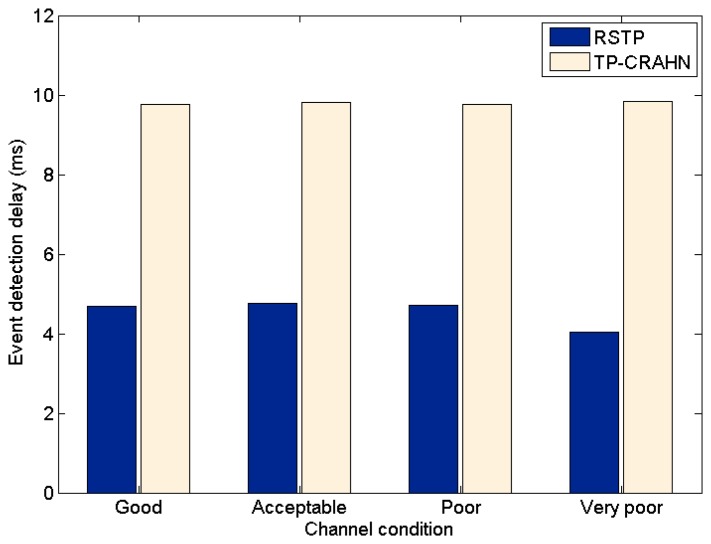
Event detection delay against channel condition.

**Figure 7.**
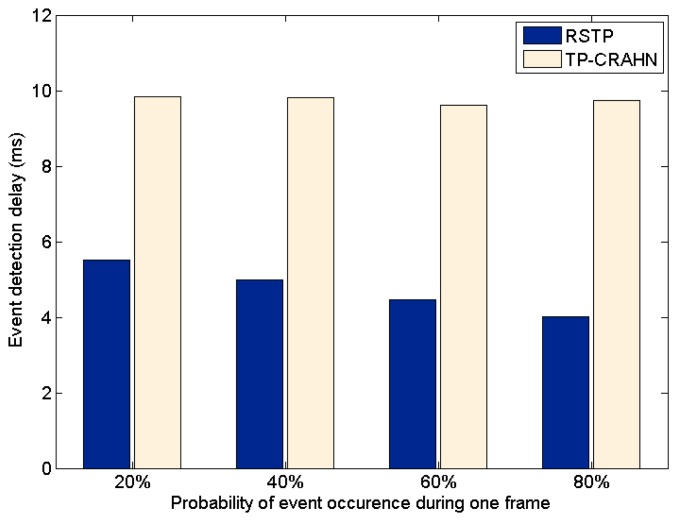
Event detection delay against probability of event occurrence.

**Figure 8.**
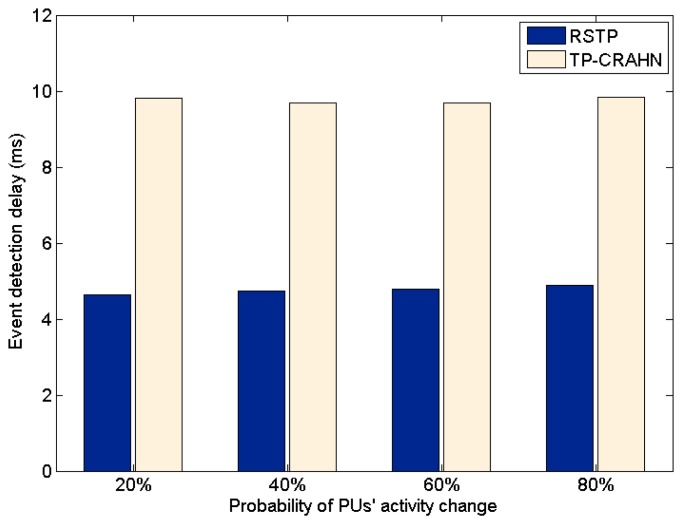
Event detection delay against probability of PUs' activity change.

Delay in event detection is defined as the time elapsed between the occurrence of a real event and the detection of the event by the sink. Figures 6, 7 and 8 show the event detection reliability in three scenarios: varied channel condition, varied probability of event occurrence, and varied probability of PUs' channel change, respectively.

The channel condition is varied from good to very poor, representing the packet error probability. The probability of occurrence of an event is varied from 20% to 80%. The PUs' channel change is defined as the occasion of PUs changing their operating channel, and it is varied from 20% to 80%. For all settings, the simulation results show the same trend; RETP has shorter delay than TP-CRAHN by 53.52%, 51.18%, and 51.33% in the scenarios of varied channel condition, varied probability of event occurrence, and varied probability of PUs' channel change, respectively. These simulation results show that even though the performance of each of the two protocols is stable under these varying conditions, RETP always has the shorter delay. The reason is, in RETP, data transmission activities are scheduled by the sink. Therefore, if the data is transmitted successfully, then the delay is fixed. On the other hand, in TP-CRAHN, data transmission occurs in a sporadic manner, resulting in inconsistent and longer delays (in the case of route failure).

### 3.4. Reliability of Event Detection

**Figure 9.**
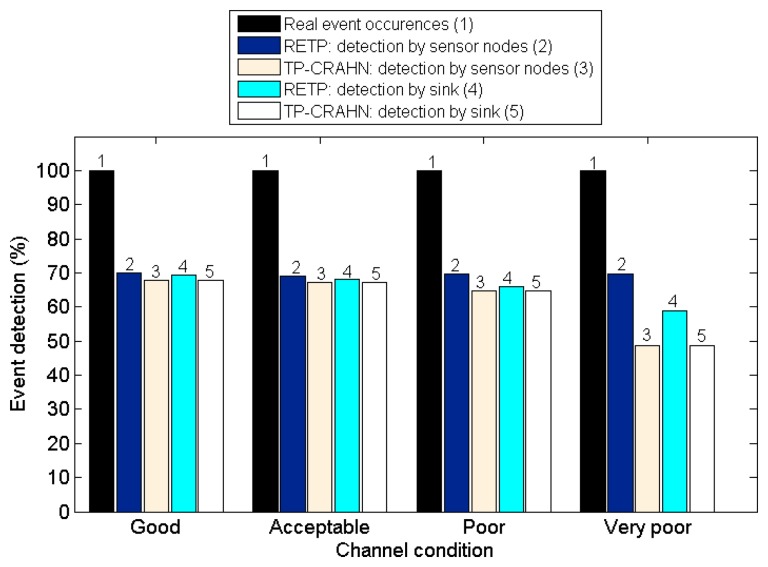
Event detection probability against channel condition.

**Figure 10.**
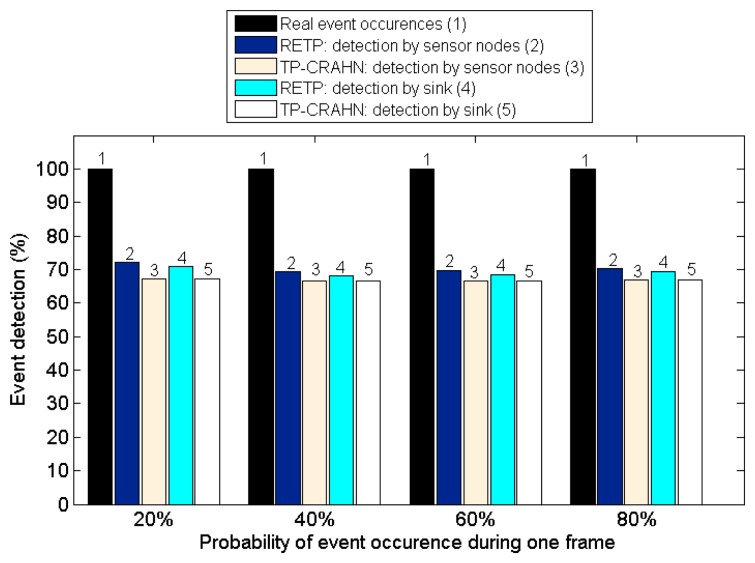
Event detection probability against probability of event occurrence.

**Figure 11.**
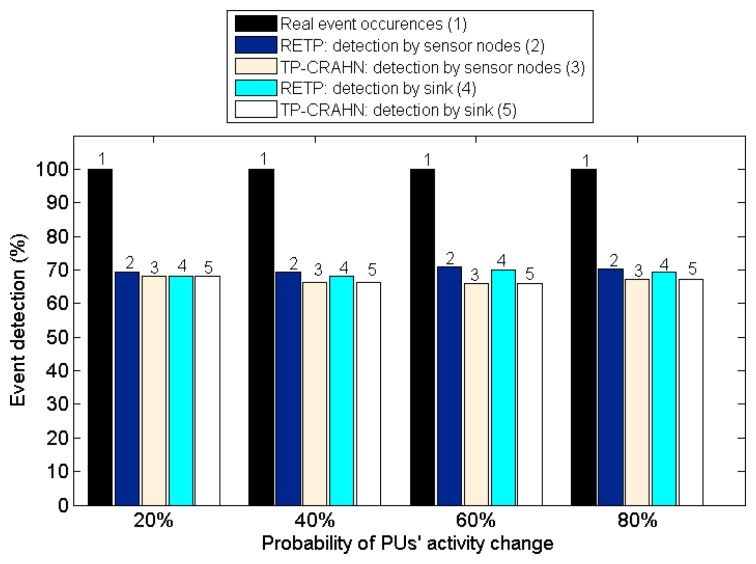
Event detection probability against probability of PUs' activity change.

High reliability of event detection is one of the most important requirements of a sensor network. The reliability of event detection is the capability of the sensor nodes to detect the occurrence of a real event and deliver the information successfully to the sink. In other words, the sink needs to be informed about every event detection. When an event such as a rise in the temperature occurs, a sensor node is able to sense the event if it is inside the sensor node's sensing coverage limit. It is possible that an event is sensed by more than one sensor node. In this case, the sensor node creates a package containing the event information and sends it to the sink. Sometimes, if the quality of the channel is poor, data transmission from the sensor node to the sink might fail. In this case, the event is detected by the sensor node, but it is not detected by the sink, which makes the CRSN fail to deliver the data to the users. Figures 9, 10 and 11 show the event detection reliability in three situations: varied channel condition, varied probability of event occurrence, and varied probability of PUs' channel change, respectively.

Figure 9 shows the event detection probability against the channel condition. The real event occurrence is the actual event simulated; thus its value is 100%. The event is directly detected by the sensor nodes (detection by sensor nodes) and the sensor nodes send the data to the sink (detection by the sink). The simulation results show that not all the events are detected by the sensor nodes. Indeed, the highest detection rate by the sensor nodes is 69.85%. Furthermore, in most cases, not every detection by the sensor nodes is successfully informed to the sink, *i.e.*, the detection by the sink is almost always lower than the detection by sensor nodes. The detection by sink is varied from 48.66% to 69.37%, relative to the real event occurrence, or from 84.38% to 100% relative to the event detection by sensor nodes. The reason that the sensor nodes fail to detect an event is that they are occupied with other tasks such as spectrum sensing and spectrum management. Nevertheless, the detection by sensor nodes in RETP remains stable (average 69.58%) whereas it decreases in TP-CRAHN from 67.75% to 48.67% as the channel quality degrades. The detection by sensor nodes in RETP is higher than in TP-CRAHN by a minimum of 2.84% when the channel condition is acceptable and a maximum of 30.28% when the channel condition is very poor. These results show that the performance of RETP remains stable even if the channel condition becomes worse.

Some events detected by sensor nodes might not be successfully received by the sink. Thus, the rate of event detection by the sink is at most the same as that of detection by sensor nodes. When both the rates are same, it means that every data transmission from sensor nodes to the sink is successful. Naturally, as the channel condition becomes worse, the rate of detection by the sink would become lower than that by sensor nodes, because the probability that the data transmission is lost or erroneously received becomes higher. The rate of detection by the sink relative to that by sensor nodes in RETP is 94.20% on average, whereas in TP-CRAHN, it is 99.98% on average. TP-CRAHN shows better performance compared to RETP by 5.78%. The reason is, in RETP, for each event detection, a sensor node sends a SDATA package and waits for an ACK. If it fails to receive an ACK, it retransmits the data on the next schedule during the same frame. When the current frame ends, the sensor node starts the next frame by following the operation mode and ignores its pending SDATA, making the sink fail to detect the event. In TP-CRAHN, the network activities are not divided in frames and the sensor nodes do not follow a centralized schedule; therefore, when an event occurs, the sensor nodes send this data to the sink immediately and the route failure method is ready to repair link failures.

Figure 10 shows the event detection probability against event occurrence probability. The results show that both protocols perform satisfactorily under these variations. Overall, RETP outperforms TP-CRAHN by 3.00% and 1.88% for detection by sensor nodes and detection by sink, respectively. These results show that both protocols are able to handle frequent event occurrences. Figure 11 shows the event detection probability against PUs' channel change probability. Similar to previous results, the performance of both protocols remain stable, even though the probability of PUs' activity change increases. Overall, RETP outperforms TP-CRAHN by 3.61% and 2.53% for detection by sensor nodes and detection by sink, respectively. This result shows that both protocols are able to adapt to frequent PUs' activity changes. Table 2 quantitatively summarizes the improvement of the performance metrics measured.

## 4. Conclusions

In this paper, a transport protocol for CRSNs called the robust and energy-efficient transport protocol has been proposed. RETP focuses on prolonging the network lifetime of CRSNs while simultaneously reducing event detection delay and maintaining reliability. The protocol operates in two modes: management mode and data collection mode. In RETP, channel sensing and channel decision are performed in a distributive manner by the sensor nodes, whereas data transmission is governed by the sink. The sink broadcasts a schedule for each frame in which it is followed by the sensor nodes. RETP has two types of data. SDATA has to be transmitted immediately for which an acknowledgment from the sink is required. RDATA does not require an acknowledgement from the sink, but if the sink does not receive any data, it sends a NACK packet. We have evaluated the performance of RETP and compared it with the performance of TP-CRAHN. Simulation results show that RETP achieves 53.8% longer network lifetime compared to that of TP-CRAHN while achieving shorter event detection delay and preserving stable event detection probability. In future works, we plan to exploit the common control channel that is idle during data collection to facilitate urgent notifications such as that for a PU's appearance on the operating channel.

## Figures and Tables

**Table 1.** Simulation settings.

**Parameter**	**Value**
Topology	50 m × 30 m × 7 m
Number of sensor nodes	60
Number of primary users	6
Number of sink nodes	1
Locations of sink node	(25 m, 15 m, 3.5 m)
Sensing range (environment sensing)	10 m
Number of channels	30
Number of timeslots per frame	100
Duration of a timeslot	20 ms
Power supply	2 AA alkaline battery (23,760 J)
Transmit power (initial)	459 μJ
Beacon power	45.9 μJ
Receive power	378 μJ
Active power	432 μJ
Idle power	172.8 μJ
Sleep power	540 nJ
Sensing environment power	1031.4 μJ
Spectrum sensing power	236.9 μJ
Configuration power	207.29 μJ
Spectrum switching power	296.13 μJ

**Table 2.** Summary of performance improvement.

**Performance Metric**	**Improvement of RETP to TP-CRAHN**	**Average Improvement**
Lifetime	53.77%	53.77%

Delay in event detection	16.56%	7.72%
	3.00%	
	3.61%	

Reliability in event detection	5.78%	3.40%
	1.88%	
	2.53%	
